# Talking about Sexuality in Stroke Individuals: The New Era of Sexual Rehabilitation

**DOI:** 10.3390/jcm12123988

**Published:** 2023-06-12

**Authors:** Marianna Contrada, Antonio Cerasa, Caterina Pucci, Irene Ciancarelli, Giovanni Pioggia, Paolo Tonin, Rocco Salvatore Calabrò

**Affiliations:** 1 S. Anna Institute, Via Siris 11, 88900 Crotone, Italy; 2 Institute for Biomedical Research and Innovation (IRIB), National Research Council of Italy, 98164 Messina, Italy; 3 Pharmacotechnology Documentation and Transfer Unit, Preclinical and Translational Pharmacology, Department of Pharmacy, Health Science and Nutrition, University of Calabria, 87036 Rende, Italy; 4 Department of Life, Health and Environmental Sciences, University of L’Aquila, 67100 L’Aquila, Italy; 5 IRCCS Bonino-Pulejo, V.le Europa, 45, 98124 Messina, Italy

**Keywords:** stroke, sexual dysfunction, holistic intervention, rehabilitation

## Abstract

One of the largest causes of mortality and disability worldwide is stroke. In the last twenty years significant objectives have been achieved in the early and chronic treatment of motor and cognitive dysfunctions, increasing the quality of life in patients and their caregivers. However, there is an unresolved clinical issue that remains: sexual dysfunctions. Multiple etiologies, including organic (such as lesion localization, premorbid medical problems, and drugs) and psychosocial (such as fear of recurrences, loss of self-esteem, role shifts, anxiety, and depression), are associated with sexual deficits. In this perspective review, we reported the last piece of evidence about this crucial topic which drastically affects the quality of life of these patients. Indeed, although patients may often not disclose their sexual concerns, literature demonstrates that they seek help concerning this issue. On the other side, clinicians working in the rehabilitation field are not always comfortable or prepared to deal with sexuality and sexual function in neurological patients. A new phase of the training course should be launched including different physicians, nurses, rehabilitation specialists, and social workers, to learn how to deal with topics related to sexuality. As a result, professional sexual counselors should now become a structured part of stroke settings and rehabilitation with new effective tools (i.e., PLISSIT model; TDF program) for improving quality of life.

## 1. Introduction

In 2002, the World Health Organization [1] defined sexuality as “… a central aspect of being human throughout life encompasses sex, gender identities and roles, sexual orientation, eroticism, pleasure, intimacy and reproduction”, and this means that sexual well-being should be guaranteed to all human beings, including those with physical and intellectual disability. “Sexuality is experienced and expressed in thoughts, fantasies, desires, beliefs, attitudes, values, behaviours, practices, roles and relationships. While sexuality can include all of these dimensions, not all of them are always experienced or expressed. Sexuality is influenced by the interaction of biological, psychological, social, economic, political, cultural, legal, historical, religious and spiritual factors. [2]”. This is why clinicians dealing with sexual dysfunction, especially in patients with neurological disorders, should have a broad knowledge of neuroanatomy and physiology as well as psychological and socio-relational issues involved in human sexuality and sexual function.

After 20 years, nowadays sexuality in stroke patients is still considered taboo by patients themselves, caregivers, and mainly healthcare professionals [3]. In the last ten years, many initiatives and a lot of new evidence have been published demonstrating the pivotal role of sexual function restoration to be as crucial to functional recovery as any other feature in the context of rehabilitation [4,5,6]. In a recent epidemiological study, Bugnicourt et al. [7] demonstrated that one year after an ischemic stroke, over one-third of younger patients reported having difficulty engaging in sexual activity. The rates of sexual dysfunctions (SDs) after stroke in literature are reported as between 20 and 75% [3,4,5,6,7,8]. Such a wide range can be explained by: (a) the choice of different inclusion and exclusion criteria in the various studies; (b) different designs of the clinical studies [8]; and (c) the different degrees of disability in patients after stroke.

SDs typically entail libido, erection, sexual arousal, and orgasm, as well as frequency of sexual activity [3,4,5,6,7,8]. Following a stroke, libido is most frequently lowered, and the prevalence of decreased sexual desire has been reported as between 17% and 42% [9]. Furthermore, significantly less sexual activity occurs as well: 28% and 14% after two and six months after the stroke, respectively [3,4,5,6,7,8,9]. The prevalence of erectile dysfunction in these patients is significantly elevated with respect to that of the general population in Europe (up to 50% vs. 19%) [9]. At a sociodemographic level, aging, low income and high education are additional factors that can influence the emergence of SDs in patients with stroke, while gender is not decisive in its development [4,10].

At neurological levels, SDs in post-stroke patients are commonly due to the damage to the central nervous system areas controlling sexual behavior, and the autonomic system determining erectile dysfunction. For example, strokes in the right cerebellum territory might be associated with ejaculation disorders, while erectile dysfunction may be more frequent in patients with stroke in the middle cerebral artery territory, as in the right hemisphere versus left hemisphere (87.5% vs. 70.6%) [11,12].

In addition to organic factors, post-stroke SDs may also be influenced by psychological issues (such as anxiety or mood disorders), prior medical conditions (such as hypertension or diabetes mellitus), or the use of particular medications to address these issues. It has been shown that rather than the actual stroke itself, the key factors related to post-stroke impaired sexual activity were angiotensin-converting enzyme (ACE) inhibitors, diabetes mellitus, and depression [6,11,12]. Despite this clinical evidence, the rehabilitation team often ignores sexual function in patients with neurological illnesses, such as stroke, as healthcare professionals tend to think that the motor-cognitive problems that brought the patient to their attention are more significant. Instead, the various physical, psychological, and behavioral changes that follow a neurological disease should be also addressed in terms of their potential to impair sexual function during both the acute and long-term rehabilitation phases [13]. As recently demonstrated by Low et al. [3], only 23% (*n* = 216 out of 958) of stroke rehabilitation professionals directly initiate sexual dialogues with their patients. Notably, they demonstrated that sexuality education, religious affiliation, age of professionals, and availability of programs for sexuality rehabilitation predicted comfort in addressing this issue.

Information about sexual activity after a stroke is yet another area that rehabilitation facilities severely undervalue. Over 30% of participants wished to receive information about post-stroke sexual behavior, but only a small percentage of participants (8.2%) received it, as shown by Prior et al. [14] when they investigated 1265 stroke patients during the rehabilitation period. These authors focused on the significance of creating fresh post-stroke information and teaching processes throughout a typical inpatient time. As illustrated by Low et al. [3], healthcare workers may not feel comfortable bringing up sexual matters owing to a lack of education, religious convictions, or training. Whenever sex and disability are discussed during the counseling of a neurological patient, it is solely in terms of capacity, technique, and fertility, and there is no reference to sexual feelings. Although it is still ignored and underreported, it is crucial to identify the obstacles that neurological patients face when trying to get assistance for sexual difficulties. According to patients, the dominance of neurological symptoms, the presence of family or friends during appointments, and not being asked are the most frequent barriers to discussing sexual concerns [3,15]. A key barrier preventing people with disabilities from exploring their sexuality is embedded social attitudes [16].

Up to today, few reviews, meta-analyses, and epidemiological studies have been carried out on the sexual dimension of post-stroke patients, assessing other aspects of sexuality such as self-esteem, gender, identity of sexual orientation, reproduction, intimacy, eroticism, and sexual communication (physical and psychological aspects of sexual functioning). For this reason, clinicians are underprepared to address sexuality with stroke patients [15,16] and rarely get informed about sexuality [17,18]. Thus, it is relevant to clearly define sexual dimension deficits affecting post-stroke individuals, as well as new programs intended to optimize sexual rehabilitation services.

In this perspective paper we propose a comprehensive conceptual framework for sexuality and chronic illness. Two fundamental components of sexuality are discussed in this model: sexual functioning, which refers to the physiological aspects of sexual performance (such as sexual arousal, orgasm, and drug treatments), and sexual well-being, which refers to a person’s subjective experience of sexuality (e.g., satisfaction, esteem, perceived sexual appeal). This perspective review is composed by three main parts: (I) sexual function and its correlates, including neurological underpinning of post-stroke SD, post-stroke disability and comorbidities as well as pharmacological treatments potentially affecting sexual function; (II) a biopsychosocial approach to post-stroke SD with currently most used and suggested assessment tools; and (III) an evidence-based discussion on the importance of assessing sexuality in the rehabilitation field with the author’s point of view and future perspective.

## 2. Neurophysiological Correlates of Sexual Dysfunction in Post-Stroke Patients

As stroke may involve different neuroanatomical areas and circuits subtending human sexual behavior and function, patients typically present with pain during sexual engagement, libido loss, erectile and/or ejaculatory dysfunctions, and anorgasmia. [19]. Concerning gender differences, spasticity, bladder incontinence and aphasia affect sexual function more frequently in females, whereas severe hemisyndrome and behavioral disorders are the main risk factor for male SD [11,14,18]. Loss of libido is a frequent and poorly investigated SD in both sexes; erectile dysfunction is the main concern of male stroke patients whereas anorgasmia is the most frequent SD in females.

Generally, ischemic lesions into specific cortical areas (such as the insula, limbic/hypothalamic regions and brainstem nuclei) are often associated with the occurrence of SDs. Table 1 summarizes the neuroanatomical basis of the main SDs.

Although a loss of libido and hyposexuality are very common SDs following stroke, hypersexuality represents the most challenging problem whenever it occurs. Indeed, in some cases, hypersexuality may be associated with deviant sexual behavior and other inappropriate social behaviors. The presence of hypersexuality with increased libido and coital frequency, as well as sexual deviations, has been correlated with lesions of limbic structures, especially in the temporal lobes, while the possible involvement of the basal ganglia has rarely been documented [20,21].

Erectile dysfunction (ED) is another common problem in stroke patients, although the etiology is often multifactorial involving neurological, vascular, hormonal, and psychogenic causes, often combined in different percentages. Winder et al. [22] carried out an interesting study in 52 male patients with a stroke to investigate the association between ED and the post-stroke ischemic lesion site using a voxel-based lesion mapping. They found that ED was associated with lesions in the right occipito-parietal and thalamic areas, which are known to integrate visual and somatosensory information. Moreover, lesions involving the left insular and parieto-temporal areas may contribute to ED, as they generate visceral arousal states.

Physical impairment has been also recognized as an important cause of long-term sexual disorders. In fact, severe stroke may influence body positioning and movement and affect the ability to properly engage in sexual intercourse. Patients might become unable to participate in physical contact (such as embracing and stimulating), have drooling, bladder and bowel incontinence, and other unwanted and inappropriate behaviors [4,6]. Moreover, facial palsy, memory and other cognitive deficits, severe hemiparesis with spasticity and hemihypoesthesia may further prevent such patients from having a “normal” sexual life. Again, stroke patients with aphasia frequently present difficulties in their sex lives. In fact, deficits in speaking, understanding, and communicating cause severe impairment in sexual relationships; moreover, in these patients it is obviously more difficult to recognize the need to treat the problems of sexual life. Indeed, sexual non-communication may become more of a problem for the partners [23]. Therefore, healthcare professionals should always inquire about any sexual problems in patients with aphasia, during the rehabilitative path.

Once more, a general and urogenital examination is required to identify medical comorbidities, particularly in stroke patients. Actually, ED can be the initial clinical symptom of an undiagnosed, untreated cardiovascular disease, so it is important to accurately assess the heart and major arteries in some people. In fact, many risk factors for stroke, including diabetes, hypertension, and dyslipidemia, may also affect ED, and vice-versa ED may be a first sign of atherosclerosis and then a potential marker of stroke. Indeed, in a recent meta-analysis, Zhao and colleagues demonstrated that the presence of ED increased the rate of coronary heart disease by 43–59% [24]. Then, managing the most common risk factors for stroke may reduce the severity and progression of ED.

Finally, one should bear in mind that several medications often prescribed to neurological patients, such as antidepressants, neuroleptics, sedatives, beta-blockers, and diuretics, can cause SDs through different and often poorly understood mechanisms. In detail, antidepressants are commonly used in the neurorehabilitation of stroke to treat depression and pain. Antidepressant-related SDs seem to be due to an imbalance of brain serotonin/dopamine ratio, where dopamine is fundamental to sexual desire and pleasure while serotonin negatively affects sexuality also by delaying ejaculation/orgasm time [25]. In fact, SSRIs are drugs with a higher prevalence of SDs, including delayed ejaculation and anorgasmia. On the contrary, SNRIs (with regard to the multimodal compound vortioxetine) and bupropion are known to cause SDs less frequently [26].

Moreover, since stroke may also occur at a young age, particular attention should be paid to this patient population when using SSRIs because of the potential onset of the still-poorly investigated post-SSRI syndrome. In fact, patients may show SDs, with regard to genital anesthesia and anorgasmia, as well as emotional blunting and mental fog for months following drug withdrawal [27]. Unfortunately, the syndrome is still under-recognized and patients do not receive the due attention and treatment.

## 3. A Biopsychosocial Perspective: Toward a Standardized Evaluation?

Biopsychosocial perspective has been suggested as the greatest comprehensive strategy for figuring out how physical, neurophysiological, psychological, and social elements influence sexuality [28]. In order to account for the complexity of stroke patients’ well-being and life satisfaction, here we provide evidence sustaining this new perspective about SDs in stroke patients.

Starting from a biological level, SDs should be first evaluated considering sex hormone concentrations regulating sexual activity. These hormones control desire, mood, and sexual functions, which are essential elements of both women’s and men’s sexuality. Sex steroids also play a role in the post-stroke healing phenomenon in addition to their previously mentioned roles. Consequently, the various steroid hormone concentrations that distinguish the two sexes could possibly be connected to the different post-stroke recovery processes in men and women. Progesterone reduces the excitatory amino acid glutamate and the generation of pro-inflammatory cytokines, lowering excitotoxicity and cell death. Estrogens have also been demonstrated to increase neurogenesis [29]. Estrogens may also cause neuronal plasticity following stroke [30]. In light of these factors, hormone replacement treatment may theoretically be regarded as helpful in stroke patients to aid in both rehabilitation and the stabilization of a destabilized sexuality. More research is necessary, though, as evidence from hormone replacement treatment in humans and rats does not show a clear conclusion [30]. As a result, a novel understanding of the function of sex hormones suggests that these steroid hormones may serve as indicators of the success of rehabilitation and post-stroke functional recovery [31].

Concerning the psychological aspects, both personality and mood disorders as well as romantic relationship problems may affect sexuality in post-stroke SDs. The direct or indirect link with mood disorders is overlooked when evaluating sexuality in stroke patients. Indeed, as demonstrated by Meesters et al. [32], sexuality and relationship satisfaction may be impacted by anxiety and mood disorders. Depression, anxiety, and post-traumatic stress disorder are psychiatric symptoms often occurring following a stroke. Post-stroke depression seems to be related to a worse prognosis, including more dependence in activities of daily living and higher mortality [33]. At the same time, this may affect sexual function with an important worsening of the quality of life of both the patients and caregivers [34]. There is a bidirectional correlation between sexuality and depression. Among the general population, subjects affected by depression may develop a 70% increased risk of developing SDs; on the other hand, individuals with SDs may have up to 210% increased risk of having depression. Indeed, according to Atlantis and Sullivan [35], both SDs and depression reflect failures of common functional systems (mainly involving the dopamine and serotonin neurotransmitters), leading to pathological feedback cycles. These pathways are often impaired following stroke, and this is why both depression and SDs are so common after brain injury. Moreover, as previously stated, the treatment of depression itself may cause iatrogenic SDs. In order to rule out any potential psychological or psychiatric factors behind SDs in these patients, a psychological screening for depression and anxiety disorders should always be conducted. Concerning romantic relationships, motor disability as well as cognitive and behavioral problems cause changes in the relationship since the partner often becomes the patient’s “caregiver” [34]. Then, this may affect sexual intimacy since the patients may feel less attractive and the partner, from his/her side, may lose sexual interest. In this vein, the psychological distress and burden of caregivers are relevant, although this issue has been poorly investigated. A study by Korpelainen et al. [11] found that a decline in libido, coital frequency, and sexual arousal as well as satisfaction was associated with various psychological factors, including attitude toward sexuality, capacity to discuss sexuality, and fear of stroke recurrence during sexual intercourse.

Sexual dysfunctions per se do not necessarily produce distress; therefore, they cannot explain sexual well-being on their own, even though identifying predictors for SDs is of the highest relevance for clinical purposes and sexual well-being [36]. Likewise, having an unsatisfactory sexual life does not necessarily indicate that there are SDs but may be due to other circumstances. Stemming from this new perspective, the concept of satisfaction in sexual life is recognized as a key aspect of well-being [37]. Investigating sexual life satisfaction enables us to reveal the distinct subjective experiences of sexual concerns beyond sexual function and recognizing the variety of elements that may influence sexual pleasure after stroke. Preliminary psychosocial studies reported that unwillingness for sex, general attitude toward sexuality, inability to discuss sexuality, and a belief in an adverse effect of stroke were explanatory factors for decreased sexual satisfaction [11,38]. In a recent study, using a multivariate biopsychosocial approach, Vikan and colleagues [39] demonstrated that partner rejection and a decrease in sexual activity had a significant contribution to sexual dissatisfaction in stroke patients. In the realm of socio-demographic research, other factors should be taken into account when evaluating sexuality in stroke patients. Overall, social resources (an aggregation of terms such as social network, participation, and perceived support) have been demonstrated to act as protectors against an adverse prognosis. In particular, Nakagawa et al., [40] recently found that prior to their stroke, patients who were more active in social groups showed less functional decline after their stroke than those who were less active. The data also showed that people who maintained contact frequently from pre-to-post-stroke had less pronounced functional deterioration over time. Social resources also affect sexuality in stroke patients. Indeed, Vikan and colleagues [39] reported that affectionate support was the only factor that contributed to sexual satisfaction, demonstrating that having someone show you love and affection is crucial for total sexual fulfillment.

In a future biopsychosocial perspective, the employment of standardized measurements for assessing biopsychosocial factors associated with SDs is necessary [41,42]. Unfortunately, there are few validated, user-friendly scales available. Indeed, no specific questionnaire, as far as we know, has been validated to properly investigate sexual life and sexual function in patients with neurological disorders, including stroke. Many scales investigate the degree of disability and quality of life following stroke, but none of them addresses (at least partially) SDs. For example, the Stroke Impact Scale evaluates how stroke has impacted a patient’s health and quality of life with items on mobility, cognition, mood, social activities, with no question on sexual function [43].

(1) To investigate sexual functioning in individuals with stroke, the following tests can be used:
  (i) Arizona Sexual Experience Scale (ASEX): a brief five-item scale assessing the core elements of sexual function (i.e., drive, arousal, penile erection/vaginal lubrication, ability to reach orgasm, and satisfaction with orgasm), [44]. ASEX is a very useful international tool to assess sexual dysfunction in people suffering from mental illness; is a well-validated and handy self-report test that was originally applied in patients receiving antidepressant drugs. ASEX is composed of five items, assessing sex drive, arousal, vaginal lubrication/penile erection, ability to reach orgasm, and orgasm satisfaction. Sexual dysfunction is defined as: (1) a total ASEX score of ≥19; (2) any item with a score of ≥5; or (3) any three items with a score of ≥4.
  (ii) Female Sexual Function Index (FSFI) [45]: a quick questionnaire-style measure of female sexual health created specifically for assessing domains of sexual function, or (iii) International Index of Erectile Function (IIEF) [46], a standardized and validated 15-item self-evaluation scale that provides pre–post-treatment clinical evaluations of erectile function, orgasmic function, sexual desire, satisfaction with sexual intercourse, and general satisfaction;
  (iii) Sexuality Evaluation Schedule Assessment Monitoring (SESAMO) [47] is useful for assessing post-stroke relational problems. This questionnaire provides the clinician with an individual’s sexual and socio-affective profile, aiming to identify any dysfunctional aspect in the individual’s and/or couple’s sexuality reporting. It consists of two forms, male and female, divided into three sections: Section 1 (General Part) provides general information; Section 2 (Single) allows describing of the situation as a single; and Section 3 (Couple) provides information for couples.
  (iv) Finally, another questionnaire that should be applied to stroke patients is the Physical Disability Sexual and Body Esteem (PDSBE) [48]. The PDSBE has been validated in patients with physical disabilities but never on stroke patients. It was designed to gauge respondents’ ability to feel confident about their sexuality and physical appearance despite having a physical disability. The elements were body esteem, sexual esteem, and attractiveness to others.
(2) To investigate sexual satisfaction in individuals with stroke, ASEX can be also employed since items for orgasm satisfaction have been included. Moreover, additional tests could be as follows:
  (i) LiSat-11: Life Satisfaction Checklist (that includes sexual satisfaction), a measure of life satisfaction, evaluating important life domains such as vocational, financial, and leisure situations, contacts with friends, self-care management, sexual life, and partner relationships.
  (ii) Maudsley Marital Questionnaire (MMQ-rs) is used to assess relational satisfaction and, even if is not specifically validated for patients with stroke, represents a valid evaluation tool to investigate couples’ sexual life.
  (iii) Pinney Sexual Satisfaction Inventory (PSSI) is composed of 51 items that address a wide range of sexual issues. For example, items were designed to inquire about the amount and quality of time before, during, and after lovemaking; frequency and satisfaction with specific sexual behaviors such as foreplay, cunnilingus, fellatio, and orgasm; whether the respondent’s partner was sensitive and responsive; and if communication was open.
  (iv) Index of Sexual Satisfaction (ISS): This scale consists of 25 items that are rated on a 5-point scale ranging from rarely or none of the time, to most or all of the time. Sample items are “My sex life is very exciting” and “My partner does not satisfy me sexually.”
  (v) Sexual Interaction Inventory (SII): this instrument was designed to assess sexual functioning and sexual satisfaction in couples. However, the use of the SII is limited to the assessment of couples when both partners are available for assessment. Another disadvantage to this scale is that it is composed of 102 items, making it time-consuming to complete.
(3) Finally, in addition to the conventional age, gender, relationship status, educational attainment, occupation, and financial-position scales, the following additional scales can be taken into account in the psychosocial and sociodemographic assessments:
  (i) Hospital Anxiety and Depression Scale (HADS): this is a validated self-assessment scale indicating depressive and anxiety symptoms, often used for the assessment of psychological factors in patients with sexual dysfunctions.
  (ii) Medical Outcomes Study-Social Support Survey (MOS-SSS): this is used to assess perceived functional social support. The scale includes four subscales with 19 items altogether (emotional/informational support, tangible support, affectionate support and positive social interaction). Response alternatives are rated on a 5-point Likert scale, with higher values indicating a higher level of support.
  (iii) Sexual Complaint Screener (SCS), with separate versions for women (SCS-W) and men (SCS-M), is used for self-reported sexual complaints per se after stroke. Response options ranged from 0 (occurring never/almost never) to 4 (occurring almost all the time/always). Personal distress related to each of the complaints during the last 6 months was reported ranging from 0 (not at all a problem) to 4 (a very great problem). Finally, the SCS includes a question on wishes for follow-up consultation for sexual problems (no vs. yes).

## 4. Discussion and Future Perspectives

Talking about and treating sexual issues in stroke patients enters the framework of a holistic approach, reminding us that invisible impairment (hidden disabilities), or disabilities that are not immediately apparent are chronic conditions that seriously affect normal daily activities. According to Ek et al. [49], people who additionally experienced SD more frequently showed repercussions that may be unnoticeable to others. A person who has an invisible handicap may seek to limit disclosure by staying away from social situations. This restriction could lead to a vicious loop in which people hide their problems from medical professionals, who then fail to recognize the person’s challenges.

Although physicians do not often ask about SDs in the rehabilitation setting, patients with stroke need the problem to be addressed [50]. Indeed, they may consider SD a more severe symptom than the motor/cognitive problems that brought them to the rehabilitation team. Sometimes, barriers such as false myths and beliefs on sexuality and disability, the presence of the partner or other family members during the visit may prevent them from spontaneously disclosing their sexual concerns [13,14]. On their side, physicians may not feel comfortable dealing with this issue, since they usually do not receive adequate training in human sexuality during their education [16]. This is why sexual medicine should enter the framework of education of every healthcare professional dealing with neurological patients, especially in the rehabilitation phase. Comprehensive sex education must include information on healthy sex and sexuality for people with neurological disabilities. Indeed, sex education should discuss intellectual and physical accommodations for such a patient population, affirming that people with disabilities are sexual beings, because they are so often seen or portrayed as desexualized or hypersexualized [51]. For anyone who has experienced changes in sexuality due to a disability, including a neurological one, and does not have a partner to work with, a sexual coach or surrogate can help develop his/her sexual potential. In detail, such a coach is “a trained professional who helps people with sexual, intimacy and relationship issues, including sexless marriage, low libido, and SD, but also guides their clients to fully grasp their sexual potential through education, training and communication”. Thanks to the expertise and the constant supervision of a therapist, sexual coaching may be really helpful to overcome SDs in people with different neurological and psychiatric disabilities [52]. Nowadays, sexual counselors can assist patients in a useful way by utilizing modern, validated models. One of the most famous models is the Permission, Limited Information, Specific Suggestion, and Intensive Therapy (PLISSIT). In the discipline of sexology, the PLISSIT model, commonly referred to as the PLISSIT model of sex therapy [53], is a modeling method used to determine the various degrees of intervention for clinical patients. Jack S. Annon constructed the model in 1976. Permission, Limited Information, Specific Suggestions, and Intensive Therapy are the four levels of intervention that a sexologist can use. Researchers demonstrate the effectiveness of sexual in-person counseling based on the PLISSIT model in several clinical domains (i.e., cancer, heart surgery) [54], although in the stroke domain it has never been employed.

A valuable example of a holistic approach to better deal with sexuality after stroke has recently been provided by Auger et al., [55]. To enhance post-stroke sexual rehabilitation services, they co-designed a theory-driven multifactorial program with stakeholders (including stroke patients, partners, doctors, managers, and researchers). They used the Theoretical Domain Framework (TDF) and the stroke core set of the International Classification of Functioning, Disability and Health (ICF) (2) in order to direct data collection and analysis at each stage of the investigation. The TDF, which has 14 different domains, was created using 33 theories about behavior change: (1) knowledge; (2) skills; (3) social/professional role and identity; (4) beliefs about capabilities; (5) optimism; (6) beliefs about consequences; (7) reinforcement; (8) intentions; (9) goals; (10) memory, attention, and decision processes; (11) environmental context and resources; (12) social influences; (13) emotions; and (14) behavioral regulation. These categories would aid implementation teams in comprehending what elements, or determinants, are most or least likely to influence practices or behaviors. Indeed, their systematic review showed that only multidisciplinary and structured interventions, including sexual counselling and pelvic-floor-muscle training, may be supported to improve sexual function in the rehabilitation setting [56]. Moreover, in a recent extensive review, the same authors aimed at identifying the assessment methods used by rehabilitation professionals to investigate sexuality in post-stroke patients [57], before any rehabilitative approach is applied. They found that SD were predominantly assessed using standardized tools (62/110 papers), while relationships and partners’ perspectives were investigated using original questionnaires as well as qualitative methods, including semistructured interviews (16 papers). The authors concluded that the best assessment methods should be the mixed one, given that bot quantitative and qualitative approaches are fundamental to properly explore sexuality in this patient population.

Another practical option is to adapt the strategies used in other neurological domains where the study regarding SD and therapy is more developed. For instance, in traumatic brain injury (TBI), many studies have been conducted. In a recent review, Robert et al. [58] demonstrated that following TBI, between 6% and 83% of patients suffer SD, including decreased frequency of sex (47–62%), decreased desire and/or arousal (24–86%), erectile dysfunction (24–57%), difficulty with orgasm (29–40%), and inappropriate sexual behavior (8–9%). SD is positively associated with mood disorders and cognitive-behavioral therapy has been proposed as the best therapeutic approach for these patients. The most recent research regarding SDs has, however, focused on patients with multiple sclerosis. Several hundred investigations have been undertaken in this field of study since the first ones were conducted in 1990. In a recent systematic review and meta-analysis, Afshar et al. [59] evaluated the beneficial effects of four kinds of treatments for improving SD: psychoeducational, exercise and rehabilitation, and medical and multi-type interventions. After interventions, more than half of psychoeducational interventions displayed a substantial improvement. Most therapies (*n* = 13/14) in the research on sexual dysfunction improved at least one subscale of sexual dysfunction. Men’s sexual dysfunction was successfully treated by medical therapies, while women’s sexual dysfunction was more successfully treated by psychoeducational interventions.

Finally, another future direction could be the entry of social robots into the sex education of stroke patients. With the advance in technology, social robots have become reliable tools for improving clinical outcomes, assisting therapists in teaching new skills or educational needs to people with different physical and intellectual disabilities [60]. In particular, sex robots are anthropomorphic mannequins with variable ages, appearances, and textures, and customizable sexual organs, which in the near future will be available for treating SD or improving sexuality also in neurological patients. Nonetheless, it has been hypothesized that sex robots’ employment would be specious without evidence-based support for their application in healthcare and rehabilitation [61].

Overall, all these interventions could therefore be positively applied to patients with stroke, given that they have proven effective in other neurological and clinical domains.

In conclusion, disregarding sexuality in disability is no longer acceptable in light of the growing recognition of the quality of life as the most significant indicator of appropriate patient management, particularly in the rehabilitation setting, and with the advent of the more effective treatment of SD. We might see a new era where sexuality is finally correctly assessed, and discussed in rehabilitation centers to educate stroke victims and their caregivers and where medical staff will be trained in coping with it. Sexual research in stroke patients should proceed along the same lines as multiple sclerosis, where a conceptual multidisciplinary framework developed from a precise and accurate diagnosis leads to a successful treatment. Sexologists, psychologists, gynecologists, and a neurologist should all be a part of the multidisciplinary rehabilitation team so that stroke patients can be assessed for sexual issues in order to build up a specific pharmacological/behavioral therapy that is also linked to rehabilitation treatment.

## Figures and Tables

**Table 1:** The relationship between the location of the lesion and sexual dysfunctions.

Brain Lesions	Sexual Dysfunction
Medial frontal cortex	Erectile dysfunction, hyposexuality
Temporal cortex	Hypersexuality
Parietal lobe Right Left inferior lobule	Altered cognitive arousal (neglect) Altered cognitive arousal (reduced sensory awareness)
Anterior cingulate cortex	Erectile dysfunction
Insula and claustrum	Altered motivational arousal Erectile dysfunction
Amygdala	Anhedonia, altered autonomic arousal Bilateral damage could lead to hypersexuality (e.g., Kluver–Bucy syndrome)
Hypothalamus	Erectile dysfunction Ejaculatory disorders (sometimes)
Thalamus	Erectile dysfunction
Nucleus accumbens	Altered motivational arousal
Left basal ganglia	Decreased libido
Mesodiencephalic tegmentum	Anhedonia
Cerebellum	Altered autonomic arousal Ejaculatory disorders Anhedonia Altered emotional arousal Ejaculatory disorders (left lesion)

## Data Availability

Not applicable.

## Abbreviations

ACE	Angiotensin-converting enzyme
SD	Sexual Dysfunction
FSFI	Female Sexual Function Index
IIEF	International Index of Erectile Function
SESAMO	Sexuality Evaluation Schedule Assessment Monitoring
PDSBE	Physical Disability Sexual and Body Esteem
TBI	traumatic brain injury

## References

[1] WHO (2002). Defining Sexual Health: Report of a TECHNICAL Consultation on Sexual Health, World Health Organization. http://www.who.int/reproductivehealth/publications/sexual_health/defining_sexual_health.pdf.

[2] WHO (2010). Sexual and Reproductive Health and Research (SRH). World Health Organization. https://www.who.int/teams/sexual-and-reproductive-health-and-research/key-areas-of-work/sexual-health/defining-sexual-health.

[3] Low M.A., Power E., McGrath M. (2022). Sexuality after Stroke: Exploring Knowledge, Attitudes, Comfort and Behaviours of Rehabilitation Professionals. Ann. Phys. Rehabil. Med..

[4] Calabrò R.S. (2022). Post-Stroke Sexual Dysfunction in Men: Epidemiology, Diagnostic Work-up, and Treatment. Innov. Clin. Neurosci..

[5] Calabrò R.S., Bramanti P. (2014). Post-Stroke Sexual Dysfunction: An Overlooked and under-Addressed Problem. Disabil. Rehabil..

[6] Calabrò R.S., Gervasi G., Bramanti P. (2011). Male Sexual Disorders Following Stroke: An Overview. Int. J. Neurosci..

[7] Bugnicourt J.-M., Hamy O., Canaple S., Lamy C., Legrand C. (2014). Impaired Sexual Activity in Young Ischaemic Stroke Patients: An Observational Study. Eur. J. Neurol..

[8] Park J.-H., Ovbiagele B., Feng W. (2015). Stroke and sexual dysfunction—A narrative review. J. Neurol. Sci..

[9] Braun M., Wassmer G., Klotz T., Reifenrath B., Mathers M., Engelmann U. (2000). Epidemiology of erectile dysfunction: Results of the ‘Cologne Male Survey’. Int. J. Impot. Res..

[10] Demir A.N., Aykan S.A., Güngör Demir U., Tunç H. (2022). Evaluation of Sexual Dysfunction in Hospitalized Post-Stroke Rehabilitation Patients. J. Contemp. Med..

[11] Korpelainen J.T., Nieminen P., Myllylä V.V. (1999). Sexual functioning among stroke patients and their spouses. Stroke.

[12] Zhao S. (2021). Significant Increase of Erectile Dysfunction in Men With Post-stroke: A Comprehensive Review. Front. Neurol..

[13] Calabrò R.S., Cerasa A., Pioggia G. (2022). When Patients Don’t Tell, Clinicians Don’t Ask: The Need for Assessing Sexuality in the Rehabilitation Setting. Ann. Phys. Rehabil. Med..

[14] Prior S., Reeves N., Peterson G., Jaffray L., Campbell S. (2019). Addressing the Gaps in Post-Stroke Sexual Activity Rehabilitation: Patient Perspectives. Healthcare.

[15] McLaughlin J., Cregan A. (2005). Sexuality in Stroke Care: A Neglected Quality of Life Issue in Stroke Rehabilitation? A Pilot Study. Sex. Disabil..

[16] Richards A., Dean R., Burgess G.H., Caird H. (2016). Sexuality after Stroke: An Exploration of Current Professional Approaches, Barriers to Providing Support and Future Directions. Disabil. Rehabil..

[17] Guo M., Bosnyak S., Bontempo T., Enns A., Fourie C., Ismail F., Lo A. (2015). Let’s Talk About Sex!-Improving Sexual Health for Patients in Stroke Rehabilitation. BMJ Open. Qual..

[18] McGrath M., Lever S., McCluskey A., Power E. (2019). How Is Sexuality after Stroke Experienced by Stroke Survivors and Partners of Stroke Survivors? A Systematic Review of Qualitative Studies. Clin. Rehabil..

[19] Calabrò R.S., Cacciola A., Bruschetta D., Milardi D., Quattrini F., Sciarrone F., la Rosa G., Bramanti P., Anastasi G. (2019). Neuroanatomy and function of human sexual behavior: A neglected or unknown issue?. Brain Behav..

[20] Monga T.N., Monga M., Raina M.S., Hardjasudarma M. (1986). Hypersexuality in Stroke. Arch. Phys. Med. Rehabil..

[21] Libman R.B., Wirkowski E.J. (1996). Hypersexuality and Stroke: A Role for the Basal Ganglia?. Cerebrovasc. Dis..

[22] Winder K., Seifert F., Köhrmann M., Crodel C., Kloska S., Dörfler A., Hösl K.M., Schwab S., Hilz M.J. (2017). Lesion mapping of stroke-related erectile dysfunction. Brain.

[23] Lemieux L., Cohen-Schneider R., Holzapfel S. (2001). Aphasia and Sexuality. Sex. Disabil..

[24] Zhao B., Hong Z., Wei Y., Yu D., Xu J., Zhang W. (2019). Erectile Dysfunction Predicts Cardiovascular Events as an Independent Risk Factor: A Systematic Review and Meta-Analysis. J. Sex. Med..

[25] Trinchieri M., Trinchieri M., Perletti G., Magri V., Stamatiou K., Cai T., Montanari E., Trinchieri A. (2021). Erectile and Ejaculatory Dysfunction Associated with Use of Psychotropic Drugs: A Systematic Review. J. Sex. Med..

[26] Chokka P.R., Hankey J.R. (2018). Assessment and management of sexual dysfunction in the context of depression. Ther. Adv. Psychopharmacol..

[27] Healy D., Bahrick A., Bak M., Barbato A., Calabrò R.S., Chubak B.M., Cosci F., Csoka A.B., D’Avanzo B., Diviccaro S. (2022). Diagnostic criteria for enduring sexual dysfunction after treatment with antidepressants, finasteride and isotretinoin. Int. J. Risk Saf. Med..

[28] Na Y., Htwe M., Rehman C.A., Palmer T., Munshi S. (2020). Sexual Dysfunction after Stroke—A Biopsychosocial Perspective. Int. J. Clin. Pract..

[29] Suzuki S., Brown C.M., Dela Cruz C.D., Yang E., Bridwell D.A., Wise P.M. (2007). Timing of Estrogen Therapy after Ovariectomy Dictates the Efficacy of Its Neuroprotective and Antiinflammatory Actions. Proc. Natl. Acad. Sci. USA.

[30] Stein D.G. (2001). Brain Damage, Sex Hormones and Recovery: A New Role for Progesterone and Estrogen?. Trends Neurosci..

[31] Haast R.A., Gustafson D.R., Kiliaan A.J. (2012). Sex Differences in Stroke. J. Cereb. Blood Flow. Metab..

[32] Meesters J.J.L., Van de Ven D., Kruijver E., Bender J., Volker W.G., Vliet Vlieland T.P.M., Goossens P.H. (2020). Counselled Patients with Stroke Still Experience Sexual and Relational Problems 1–5 Years after Stroke Rehabilitation. Sex. Disabil..

[33] Cai W., Mueller C., Li Y.J., Shen W.D., Stewart R. (2019). Post stroke depression and risk of stroke recurrence and mortality: A systematic review and meta-analysis. Ageing Res. Rev..

[34] Kautz D.D., Van Horn E.R. (2017). Sex and intimacy after stroke. Rehabil. Nurs..

[35] Atlantis E., Sullivan T. (2012). Bidirectional association between depression and sexual dysfunction: A systematic review and meta-analysis. J. Sex. Med..

[36] Velten J., Margraf J. (2017). Satisfaction guaranteed? How individual, partner, and relationship factors impact sexual satisfaction within partnerships. PLoS ONE.

[37] Sánchez-Fuentes M.D.M., Santos-Iglesias P., Sierra J.C. (2014). A systematic review of sexual satisfaction. Int. J. Clin. Health Psychol..

[38] Cheung R.T.F. (2002). Sexual functioning in chinese stroke patients with mild or no disability. Cerebrovasc. Dis..

[39] Vikan J.K., Snekkevik H., Nilsson M.I., Stanghelle J.K., Geirdal A., Fugl-Meyer K.S. (2021). Sexual Satisfaction and Associated Biopsychosocial Factors in Stroke Patients Admitted to Specialized Cognitive Rehabilitation. Sex. Med..

[40] Nakagawa T., Noguchi T., Komatsu A., Saito T. (2022). The role of social resources and trajectories of functional health following stroke. Soc. Sci. Med..

[41] Winstein C.J., Stein J., Arena R., Bates B., Cherney L.R., Cramer S.C., Deruyter F., Eng J.J., Fisher B., Harvey R.L. (2016). Guidelines for Adult Stroke Rehabilitation and Recovery: A Guideline for Healthcare Professionals from the American Heart Association/American Stroke Association. Stroke.

[42] Gustafsson L., Arfaras T. (2022). Sexuality Early after Stroke. Aust. Occup. Ther. J..

[43] Tse T., Douglas J., Lentin P., Carey L. (2013). Measuring participation after stroke: A review of frequently used tools. Arch. Phys. Med. Rehabil..

[44] McGahuey C.A., Gelenberg A.J., Laukes C.A., Moreno F.A., Delgado P.L., McKnight K.M., Manber R. (2000). The Arizona Sexual Experience Scale (ASEX): Reliability and validity. J. Sex. Marital. Ther..

[45] Wiegel M., Meston C., Rosen R. (2005). The Female Sexual Function Index (FSFI): Cross-Validation and Development of Clinical Cutoff Scores. J. Sex. Marital. Ther..

[46] Rosen R.C., Cappelleri J.C., Gendrano N. (2002). The International Index of Erectile Function (IIEF): A State-of-the-Science Review. Int. J. Impot. Res..

[47] Boccadoro L., Perillo A. (1996). SESAMO: Sexuality Evaluation Schedule Assesment Monitoring: Approccio Differenziale Al Profilo Idiografico Psicosessuale E Socioaffettivo: Manuale.

[48] Taleporos G., McCabe M.P. (2002). Development and Validation of the Physical Disability Sexual and Body Esteem Scale. Sex. Disabil..

[49] Ek A.S., Holmström C., Elmerstig E. (2023). Sexuality> 1 Year after Brain Injury Rehabilitation: A Cross-Sectional Study in Sweden. Brain Inj..

[50] Stein J., Hillinger M., Clancy C., Bishop L. (2013). Sexuality after stroke: Patient counseling preferences. Disabil. Rehabil..

[51] Schmitz M.A., Finkelstein M. (2010). Perspectives on poststroke sexual issues and rehabilitation needs. Top. Stroke Rehabil..

[52] Calabrò R.S., Pioggia G., Contrada M., Cerasa A. (2022). Sexual Coach in High-Functioning Autism: A Growing Need. Brain Sci..

[53] Annon J.S. (1976). The PLISSIT model: A proposed conceptual scheme for the behavioral treatment of sexual problems. J. Sex. Educ. Ther..

[54] Khoei E.M., Kharaghani R., Shakibazadeh E., Faghihzadeh S., Aghajani N., Korte J.E., Esmkhani M. (2022). Sexual health outcomes of PLISSIT-based counseling versus grouped sexuality education among Iranian women with breast cancer: A randomized clinical trial. Sex. Relation Ther..

[55] Auger L.P., Allegue D.R., Morales E., Thomas A., Filiatrault J., Vachon B., Rochette A. (2022). Co-designing a Program to Improve Post-stroke Sexual Rehabilitation: The Promise of Innovative Methods. Front. Rehabilit. Sci..

[56] Auger L.P., Grondin M., Aubertin M., Marois A., Filiatrault J., Rochette A. (2021). Interventions used by allied health professionals in sexual rehabilitation after stroke: A systematic review. Top. Stroke Rehabil..

[57] Auger L.P., Aubertin M., Grondin M., Auger C., Filiatrault J., Rochette A. (2022). Assessment methods in sexual rehabilitation after stroke: A scoping review for rehabilitation professionals. Disabil. Rehabil..

[58] Robert H., Pichon B., Haddad R. (2019). Dysfonctions Sexuelles Après Traumatisme Crânien: Revue Systématique de La Littérature. Prog. En. Urol..

[59] Afshar B., Amini L., Hasani M., Jahanfar S., Nabavi S.M. (2022). The Most Effective Sexual Function and Dysfunction Interventions in Individuals with Multiple Sclerosis: A Systematic Review and Meta-Analysis. Int. J. Reprod. Biomed..

[60] Blavette L., Rigaud A.S., Anzalone S.M., Kergueris C., Isabet B., Dacunha S., Pino M. (2022). A Robot-Mediated Activity Using the Nao Robot to Promote COVID-19 Precautionary Measures among Older Adults in Geriatric Facilities. Int. J. Environ. Res. Public Health.

[61] Cox-George C., Bewley S. (2018). Sex Robot: The health implications of the sex robot industry. BMJ Sex. Reprod. Health.

